# Trends and biopsychosocial correlates of physical disabilities among older men and women in Taiwan: examination based on ADL, IADL, mobility, and frailty

**DOI:** 10.1186/s12877-022-02838-6

**Published:** 2022-02-22

**Authors:** Ching-Ju Chiu, Meng-Ling Li, Chieh-Ying Chou

**Affiliations:** 1 Institute of Gerontology, College of Medicine, National Cheng Kung University, Tainan, Taiwan; 2 Department of Medicine, College of Medicine, National Cheng Kung University, Tainan, Taiwan; 3 Department of Family Medicine, National Cheng Kung University Hospital, College of Medicine, National Cheng Kung University, Tainan, 70403 Taiwan

**Keywords:** Activities of daily living (ADL), Instrumental activities of daily living (IADL), Mobility, Frailty, National Health Interview Survey

## Abstract

**Background:**

This study examines correlates of disabilities related to ADL, IADL, mobility, and frailty in men and women with a nationally representative sample of older adults living in the community.

**Methods:**

A total of 10,898 noninstitutionalized Taiwanese nationals aged 65 years and older enrolled in the 2001 (*N* = 2,064), 2005 (*N* = 2,727), 2009 (*N* = 2,904), and 2013 (*N* = 3,203) National Health Interview Survey (NHIS) were analyzed.

**Results:**

The prevalence of mobility disabilities and frailty in older adults in Taiwan decreased during the past decade (${\chi }_{Mobility}^{2}= -5.4$, ${\chi }_{Frailty}^{2}= -6.2$). Exercise, social engagement, and tea and coffee intake were found to be associated with lower levels of all types of disabilities in both men and women. In addition, a diet based on carbohydrates, falls, depressive symptomatology, lung and metabolic diseases were risks for most of the disabilities under consideration. Gender-specific independent correlates included: being married (OR = 0.63, 95%CI: 0.40–0.98), eggs/beans/fish/meat consumption (OR = 0.35, 95% CI = 0.16–0.80); depressive symptoms, obesity and cataracts, which were associated with higher IADL (OR = 3.61, 1.63, and 1.18, respectively) and frailty limitations (OR = 10.89, 1.27, and 1.20, respectively) in women. Cognitive impairment was found to be an important correlate for ADL limitations in men (OR = 3.64, 95%CI: 2.38–5.57).

**Conclusions:**

Exercise, social participation and diet (more tea and coffee intake and lower carbohydrates) were correlates for lower levels of disability. Some gender-specific correlates were also identified, including associations of disability with depressive symptoms, obesity, and cataracts that were more distinct in women, and lower levels of disability which were especially significant in men who were married, eat more eggs, beans, fish, and meat, and those free from cognitive impairment.

## Background

The population structure of Taiwan has changed from high birth and high mortality to low birth and low mortality. Currently 14.9% of the population is aged over 65, and it is estimated that the elderly population will exceed 20% of the total population by 2026 [1]. In addition to aging within the population, according to statistics from the Ministry of Health and Welfare, the number of disabled people in Taiwan in 2015 was 755,000 and is projected to increase to 1.2 million by 2031, where the proportion of disabled people among the elderly population will account for 15 to 16% [2]. With the increase in the absolute number and relative proportion of disabled individuals, expenditures on social welfare and medical assistance will increase, causing a heavy burden on society [3].

Describing the physical functions of the elderly is an important aspect of assessing senior health. At present, common scales include ADL (Activities of Daily Living, ADL) [4] and IADL (Instrumental Activities of Daily Living, IADL) [5]. IADL is used to assess the ability of an individual to live independently and requires a higher level of physical integration and cognitive functions. The ADL assessment items include self-care and mobility, so patients with lowered IADL but normal ADL results have better physical functioning than patients with ADL dysfunctions. The poorer the ADL function assessment, the higher the dependency on the caregiver [6].

Frailty and mobility assessments thus play an important role in predicting disability. According to research conducted by Gale et al. [7], the degree of functional degradation in mobility, IADL, and ADL in the frailty group is more common than that in the non-frailty group. Among indices of ADL and IADL, a higher proportion of items that require mobility are affected, including walking and getting in and out of bed in the ADL evaluation items, and shopping and housekeeping in the IADL evaluation items. Compared with a serious loss of functions, such as those applicable to ADL and IADL, frailty and upper and lower extremity functional limitations are still recoverable through interventions such as exercise [8], so it is possible to improve frailty and mobility conditions and reduce the proportion and severity of disabilities [9].

In terms of the evaluation of disability trends in various countries, the prevalence of disabled elderly individuals in the United States between 1982 and 2009 has shown a downward trend in terms of IADL disability, and ADL disability remained stable across time [10]. It was found in people over 65 years old in an American medical care beneficiary survey that the percentages of ADL, IADL, and functional disorders dropped significantly between 1992 and 1996. Even if the proportion of disabilities decreases, the fact that the number of disabilities increases as the base of the elderly population expands cannot be ignored [11].

The results of a longitudinal study (1993–2002) in Japan indicated that both ADL and IADL in the population over 66 years of age were on a significant downward trend. When the two indicators were combined, the average number of disabled people decreased by 2% a year over a 9 year period, where an increase in education level was found to be significantly related to the decline in the prevalence of disabled people [12]. The prevalence of disease and disability in different age groups is affected by gender. The prevalence of disability is higher in women than in men, especially in people at or over the age of 85. Among the elderly population between the ages of 65 and 74, stroke is the main cause of disability in men while orthopedic diseases are the main cause of disability in women. The elderly aged above 75, dementia is the main cause of disability in both men and women [13].

The purpose of this study is to investigate the trends in disability between men and women among the elderly population in Taiwan, as well as their biopsychosocial correlates, including sociodemographic factors, health condition, diet, and behavioral factors. National representative samples from the National Health Interview Survey (NHIS) in Taiwan in 2001, 2005, 2009, and 2013 are used in the present study. Four parameters were used to comprehensively assess the physical functions of the elderly, including basic ADL, functional ADL, mobility, and frailty. Understanding the trends in disability based on gender, generation, and period is an important indicator by which a country can formulate long-term care-related policies and review the effectiveness of such policies.

## Method

### Data source

Data from the National Health Interview Survey (NHIS) conducted by the Health Promotion Administration of the Ministry of Health and Welfare were used for the analyses. The National Health Research Institutes and the Health Promotion Administration of the Ministry of Health and Welfare under the Executive Yuan have established a regular National Health Interview Survey to understand the health status and need for medical care for the general public in Taiwan through regular interviews. To select the survey participants, the general household registration data for each region on January 16, 1990 was used as the sampling base, and a multistage stratified systematic sampling design was adopted, where each layer uses a sampling method that is proportional to the unit size (Probability Proportional to Size, PPS) to gradually extract “township and urban areas,” “neighborhood,” and “household” data. “Household” served as the basic sampling unit. All members of the selected households were considered to be interviewees. Data collection was conducted using a structured questionnaire and face-to-face interviews with trained interviewers. The questionnaire comprised basic demographic characteristics, household structure, living arrangement, health status and use of medical care, leisure and social engagement, emotional status, etc. The samples were representative of Taiwan in 2001, 2005, 2009, and 2013 [14]. Data for senior citizens at or over the age of 65 in 2001 (*N* = 2,064), 2005 (*N* = 2,727), 2009 (*N* = 2,904), and 2013 (*N* = 3,203) were collected with a total sample of 10,898 analyzed in the present study. The participants were all randomly selected, and respondents in separate years were not correlated. This study was performed in accordance with the relevant guidelines and regulations, including the Declaration of Helsinki and was approved by the Institution Review Board (IRB) of National Cheng Kung University Hospital in Taiwan (No. B-ER-104–077).

### Measures

Disability was assessed using the Katz Index of Independence in Basic Activities of Daily Living (ADL), the Lawton Instrumental Activities of Daily Living (IADL), Nagi’s mobility, and five frailty items. The Activities of Daily Living (ADL) [4], which includes six questions related to eating, bathing, undressing, going to the toilet, getting in and out of bed, and walking indoors. Each item was categorized as 0 or 1, and with 1 indicating a need for assistance with the given task. The total score of ADL limitations in the present study ranged from 0–6 points. Participants with ADL limitations equal to one or more were considered to have ADL disabilities. Disability on Instrumental Activities of Daily Living (IADL) [15] was defined as any difficulty related to performing the following: cooking, going out and buying things, using the phone, taking drugs, doing light housework, doing laundry, cleaning the home, and managing finances. The scores ranged from 0 to 8, where participants scoring 1 point or higher represented IADL disabilities. Mobility was assessed using five questions from Nagi’s upper and lower extremity assessment [16], including bending down / kneeling / squatting, climbing ten steps, walking 400 m, holding things with your fingers, and holding four and a half kilograms with one hand. Participants who reported having difficulty in doing any of these tasks with a score of 1 or more were considered to mobility disabled. Frailty was assessed using the Fred phenotype, which comprises five major aspects, including dullness, inability, weakness, low physical activity, and atrophy [17]. In this study, five similar questions were used, including being unable to walk 400 m, being unable to carry four and a half kilograms with one hand, holding up, lack of appetite, having problems or being unable to engage in ordinary activities. The total score ranged between 0–5, where a score of 3 or more indicated frailty.

The Mini-mental State Examination (MMSE) was used as the basis for the analysis of cognitive impairment. It tests interviewees on time, space, short-term memory, arithmetic, drawing, repetition, and following instructions, among other questions [18]. The total score of the scale ranges between 0–30 points. Then, according to the 2008 Long-Term Care Seminar in Taiwan, those with lower education level (middle school or below) with MMSE scores ≦ 17 points and those with higher education levels (high school or above) with MMSE scores ≦ 25 points were both considered to be suffering from mild cognitive impairment (MCI). The assessment of depressive symptomatology was based on the Center for Epidemiologic Studies Depression Scale (CES-D) [19]. The 10 questions included not wanting to eat, feeling bad, always having trouble, not sleeping well, feeling happy, feeling lonely, not being friendly enough, feeling good about life, feeling sad, and unable to do anything. The total score ranged from 0–30, where a score of 10 or more was considered to indicate depressive symptoms. In 2001, except for ADL, the rest of the disability cases were not included in the analysis due to limitations related to the questions (such as differences in the number of questions, definitions, etc.). The IADL in 2009 was not included in the analysis because it was different in terms of significance from the other years under consideration.

In this study, three domains of theoretically and empirically important explanatory variables related to disability, including sociodemographic [20–24], health [25–30], and lifestyle and behavioral factors [31–37], were examined. Sociodemographic variables mainly included age, gender, living alone or not, marital status, and education level. Marital status was divided into married and others (cohabiting, unmarried, divorced, widowed, separated, and others); education level was divided into low education level (below middle school, ≦ 9 years), middle education level (high school, 10–12 years), and high education level (college degree or above, 13 + years). However, due to the limitations in the questionnaire in 2001, it was impossible to judge whether the individuals in the sample lived alone or not, so this data was lacking in the 2001 sample.

Health conditions included hypertension, hyperglycemia, hyperlipidemia, heart disease, lung disease, cataracts, hearing problems, and cognitive impairment. Among them, hypertension, diabetes, and hyperlipidemia were combined into “metabolic disease,” which was defined as a diagnosis with one of these diseases by a physician. In the case of heart disease, lung disease, cataracts, and hearing problems, the questionnaire states “determined whether the subject has a disease after diagnosis by a physician.” However, since hearing problems were not diagnosed by a physician, the answer to the question “Do you wear a hearing aid?” became the criterion for having a hearing problem.

Lifestyle and health behavior included BMI, exercise, a fall within the past year, presence or absence of social engagement, depressive symptomatology, and five dietary conditions. Among them, the body mass index (BMI) referred to the definition given by the Health Promotion Administration of the Ministry of Health and Welfare [38]. If the BMI < 18.5, the respondent was considered to be underweight. If the BMI ranged from 18.5–24, the respondent’s weight was considered to be normal. If the BMI ranged from 24–27, the respondent was considered to be overweight. If the BMI ≧ 27, the respondent was considered to be obese. Social engagement comprised three topics: “Are you currently working as a volunteer?”, “Are you currently participating in religious activities?”, and “Are you currently participating in community groups or activities?” The answer options for volunteering included: never, rarely, sometimes, and often. The options for the other two questions included: no, occasionally, and regularly. If the respondent did not answer “no or never” to one of the questions, he/she was considered as having social engagement.

The five dietary scenarios included tea or coffee, eggs/beans/fish/meat, vegetables, carbohydrates, and milk and dairy products. The diet-related questions were not exactly the same in the various questionnaires. For tea or coffee, the 2001 questionnaire included two questions: “How many times do you usually drink coffee a week?” and “How many times do you usually drink tea a week?” In the 2005 questionnaire, it was, “How many times do you usually drink coffee or tea a week?” In the 2009 questionnaire, it was, “Did you have a cup (240c.c) of tea or coffee yesterday?” The 2001 and 2005 answer options included: never eat, eat less than once a week or rarely, eat 1–2 times a week, 3–5 times a week, eat every day or almost every day, etc. The answer options in 2009 were either yes or no. As long as the respondent did not answer “never eat” or “no” to one of the questions, he/she was deemed to have a diet with tea or coffee; otherwise, the respondent was not considered to be including these items in the diet. For eggs/beans/fish/meat, four questions were adopted in 2001, which were “How many times do you usually eat meat or poultry a week?”; “How often do you eat fish a week?”; “How many eggs do you usually eat a week?”, and “How many servings of beans do you usually eat a week?” The 2005 questionnaire used five questions: “How many times do you usually eat meat or poultry a week?”; “How often do you eat fish a week?”; “How many eggs do you usually eat a week?”, “How many servings of beans do you usually eat a week?” and “How many servings of fresh beans do you usually eat a week?” In the 2009 questionnaire, it was, “Did you eat half a serving of eggs, beans, fish and meat yesterday?” The 2001 and 2005 answer options included: never eat, eat less than once a week or rarely, eat 1–2 times a week, 3–5 times a week, eat every day or almost every day, etc. The answer options in 2009 were yes or no. As long as the respondent did not answer “never eat” or “no” to one of the questions, the respondent was deemed to have a diet that included eggs/beans/fish/meat: otherwise the respondent was considered to not be including these items in the diet. For vegetables, one question was adopted in 2001, which was, “How many times do you usually eat vegetables a week?” In the 2009 questionnaire, it was, “Did you eat half a serving of vegetables yesterday?” The 2001 and 2005 answer options included: never eat, eat less than once a week or rarely, eat 1–2 times a week, 3–5 times a week, eat every day or almost every day, etc. The answer options in 2009 were yes or no. As long as the respondent did not answer “never eat” or “no” to one of the questions, the respondent was considered to have a diet including vegetables; otherwise, the respondent was deemed to not be including vegetables in the diet. For carbohydrates, one question was adopted in 2001, which was, “How many bowls of rice and noodles do you eat every day?” In the 2005 questionnaire, it was, “How often do you eat rice or noodles a week?” In the 2009 questionnaire, it was “Did you eat half or more than half a serving of grains and root vegetables yesterday?” The answer options in 2001 were the actual amounts. The data range was 0–9 bowls. As long as it was greater than 0 bowls, the respondent was considered to have a diet that included carbohydrates. The answer options in 2005 included: never eat, eat less than once a week or rarely, eat 1–2 times a week, 3–5 times a week, eat every day or almost every day, etc. The answer options in 2009 were yes or no. As long as the respondent did not answer “never eat” or “no” to one of the questions, he/she was deemed to have a diet that included carbohydrates; otherwise the respondent was considered to not be including carbohydrates in the diet. For dairy products, one question was adopted in 2001, which was, “How many times do you usually drink milk (from a cow/goat) a week?” In the 2009 questionnaire, it was, “Did you drink/eat half a serving of dairy products yesterday?” The 2001 and 2005 answer options included: never drink/eat, drink/eat less than once a week or rarely, drink/eat 1–2 times a week, 3–5 times a week, drink/eat every day or almost every day, etc. The answer options in 2009 were yes or no. As long as the respondent did not answer “never eat” or “no” to one of the questions, he/she was deemed to have a diet that included dairy products: otherwise the respondent was considered to be including dairy in the diet. Finally, due to question limitations, the social engagement of the respondents could not be judged in 2001, so the data on social engagement was deficient. In 2013, since the externally released data did not include the BMI and diet-related data, a related analysis could not be conducted.

### Statistical analysis

In this study, SAS statistical software (version 9.4, SAS Institute, Inc., Cary, NC) was used. First, a trend analysis of the characteristics of the nationally representative sample of older adults in 2001, 2005, 2009, and 2013 by chi-square test with their sociodemographic, health, and behavioral variables were examined. In addition, gender differences on all the variables we examined in the present study of participants from 2001 to 2013 were also conducted using a chi-square or t-test. Further, a two way-ANOVA was also used to determine whether the gender differences in terms of disability were consistent over time. Finally, a binary logistic regression analysis was conducted to determine the factors related to disability. Models were run for each gender for each of the four disability measurements.

## Results

### Participant characteristics

Table 1 describes the sociodemographic, health and lifestyle, and behavioral characteristics of the participants over the years. The average age over the years ranged from 73.4–75.3, which has not changed significantly in terms of trend. There were more women than men in the data in most of the survey years. The proportion of women was approximately 48.8–56.9%. Over the years, nearly 60% of the respondents were married, and there has been no significant change in this trend. In terms of education level, most people had low education levels (middle or lower), accounting for 83.8–87.4%. Again, this reached statistical significance in terms of trends. Meanwhile, in each survey, men and women were statistically significantly different only in terms of marriage and education level. The men were mostly married and had high education levels, while the women showed an opposite trend. In the surveys, in terms of health status, the gender trends were as follows: metabolic disease (2001:60.0% vs. 54.8%, 2005: 62.1% vs. 54.9%, 2009:67.1% vs. 57.2%, 2013:69.4% vs. 61.5%), heart disease (2009:17.7% vs. 14.8%, 2013:21.1% vs. 18.3%), cataract (2001:37.9% vs. 25.8%, 2005: 29.9% vs. 22.8%, 2009:45.9% vs. 36.4%, 2013:40.1% vs. 28.2%), and cognitive impairment (2005: 27.0% vs 14.2%, 2009:24.8% vs. 16.3%, 2013:22.7% vs. 15.9%), where the proportion of women was significantly higher than that of men. However, in terms of lung disease (2001: 17.5% vs. 10.6%, 2005: 13.2% vs. 7.8%, 2009: 9.0% vs 6.5%, 2013: 10.9% vs 6.9%) and hearing problems (2005: 2.8% vs 1.6%, 2009: 2.5% vs 1.4%, 2013: 3.3% vs 1.5%), the proportion of men was significantly higher than that of women, respectively. In terms of healthy behavior, with the exception of social engagement and the habit of eating cereal, for which there were no significant differences between men and women over the years, most measures showed statistically significant differences between men and women over time. Among them, men tended to have normal BMI (2001: 56.0%, 2005: 48.6%, 2009: 52.0%), while women tended to be overweight and obese (2001: 45.8%, 2005: 47.6%, 2009: 50.1%). The percentage of men engaging in exercises was higher than 50% over time, which was significantly higher than women (2001: 59.9% vs. 53.9%, 2005: 57.8% vs. 50.6%, 2009: 53.8% vs. 47.2%, 2013: 53.7% vs. 46.4%). The proportion of women suffering from falls (2005: 26.0% vs 16.4%, 2009: 22.3% vs. 15.8%, 2013: 18.0% vs. 14.9%) and women with depressive symptoms (2005: 24.5% vs. 16.8%, 2009: 18.2% vs. 13.0%, 2013: 17.8% vs. 9.1%) was significantly higher than was the case for men. In terms of diet, men and women both mainly consumed cereals, vegetables, eggs, and fish, but the proportion of men who drank tea or coffee was significantly higher than that of women (2001: 61.4% vs. 39.3%, 2005: 74.2% vs. 52.3%, 2009: 50.1% vs. 30.6%), and the proportion of women who consumed dairy products was significantly higher than that of men (2001: 73.4% vs. 63.8%, 2005: 74.0% vs. 68.0%, 2009: 50.6% vs. 41.5%).

**Table 1:** Characteristics of each wave of participants (%, 2001–2013)

	Total^a^				Men^b^				Women^b^			
	2001	2005	2009	2013	2001	2005	2009	2013	2001	2005	2009	2013
Sample size	2064	2725	2904	3203	1056	1347	1253	1525	1008	1378	1651	1678
Sociodemographic												
Age												
mean (min–max)	73.40 (65–98.2)	74.03 (65–102.6)	74.75 (65–99)	75.27 (65–100.16)	73.29 (65–96.7)	73.98 (65–102.6)	75.03 (65–99.5)	75.34 (65–96.8)	73.48 (65–98.2)	74.07 (65–95.4)	74.54 (65–98.4)	75.22 (65–100.2)
Age group (years)												
65–74	65.0	59.1	55.3	48.4	65.3	**58.2**	**52.5**	53.6	64.7	**60.0**	**57.4**	53.8
75–84	30.2	34.9	35.9	41.4	30.8	**37.1**	**38.4**	35.7	29.6	**32.7**	**34.0**	36.6
85 +	4.8	6.0	8.8	10.2	4.0	**4.8**	**9.1**	10.7	5.8	**7.3**	**8.6**	9.7
Gender												
Men	**51.2**	**49.4**	**43.2**	**47.6**	-	-	-	-	-	-	-	-
Women	**48.8**	**50.6**	**56.9**	**52.4**	-	-	-	-	-	-	-	-
Marital status												
married/partnered	60.5	58.6	59.0	58.6	**74.9**	**71.6**	**73.5**	**73.6**	**54.5**	**45.9**	**48.0**	**45.0**
other	39.5	41.4	41.0	41.4	**25.1**	**28.4**	**26.5**	**26.4**	**45.5**	**54.1**	**52.0**	**55.0**
Living alone												
Yes	-	**10.1**	**12.8**	**11.1**	-	10.5	11.9	10.5	-	9.8	13.5	11.6
No	-	**89.9**	**87.2**	**89.0**	-	89.5	88.1	89.5	-	90.2	86.5	88.4
Level of education												
Low	**84.8**	**87.4**	**85.8**	**83.8**	**75.4**	**79.8**	**76.4**	**74.6**	**94.5**	**94.8**	**93.0**	**92.1**
Medium	**8.2**	**6.2**	**7.7**	**8.6**	**12.0**	**9.2**	**12.0**	**12.9**	**4.2**	**3.3**	**4.4**	**4.6**
High	**7.1**	**6.4**	**6.5**	**7.6**	**12.6**	**11.1**	**11.6**	**12.5**	**1.3**	**1.9**	**2.6**	**3.3**
Health condition												
Metabolic disease	**57.3**	**58.5**	**62.8**	**65.6**	**54.8**	**54.9**	**57.2**	**61.5**	**60.0**	**62.1**	**67.1**	**69.4**
Heart disease	**23.8**	**18.0**	**16.4**	**19.1**	23.1	17.0	**14.8**	**18.3**	24.6	19.1	**17.7**	**21.1**
Lung disease	**14.1**	**10.5**	**7.6**	**8.8**	**17.5**	**13.2**	**9.0**	**10.9**	**10.6**	**7.8**	**6.5**	**6.9**
Cataracts	**31.7**	**26.4**	**41.8**	**34.5**	**25.8**	**22.8**	**36.4**	**28.2**	**37.9**	**29.9**	**45.9**	**40.1**
Hearing impairment	2.7	2.2	1.9	2.5	3.2	**2.8**	**2.5**	**3.3**	2.0	**1.6**	**1.4**	**1.5**
Cognitive impairment	-	20.5	21.1	19.5	-	**14.2**	**16.3**	**15.9**	-	**27.0**	**24.8**	**22.7**
Behavior factors												
BMI												
Underweight	**6.5**	**6.9**	**4.7**	-	**6.5**	**6.1**	**4.7**	-	**6.5**	**7.8**	**4.7**	-
Normal	**52.5**	**46.6**	**48.3**	-	**56.0**	**48.6**	**52.0**	-	**47.7**	**44.6**	**45.3**	-
Overweight	28.0	27.4	28.2	-	**27.8**	**28.5**	**28.0**	-	**28.3**	**26.3**	**28.3**	-
Obese	**13.1**	**19.1**	**18.9**	-	**9.7**	**16.8**	**15.3**	-	**17.5**	**21.3**	**21.8**	-
Exercise	**56.9**	**54.1**	**50.1**	**49.9**	**59.9**	**57.8**	**53.8**	**53.7**	**53.9**	**50.6**	**47.2**	**46.4**
Fall	**100.0**	**21.3**	**19.5**	**16.5**	100	**16.4**	**15.8**	**14.9**	100.0	**26.0**	**22.3**	**18.0**
Social engagement	-	42.9	39.9	41.6	-	42.9	39.4	42.6	-	42.9	40.4	40.7
Depressive symptomatology	-	**20.6**	**15.9**	**13.7**	-	**16.8**	**13.0**	**9.1**	-	**24.5**	**18.2**	**17.8**
Diet												
Tea or coffee	**50.6**	**63.1**	**39.0**	-	**61.4**	**74.2**	**50.1**	-	**39.3**	**52.3**	**30.6**	-
Protein	**99.0**	**99.1**	**87.8**	-	98.7	**99.5**	**92.2**	-	99.4	**98.8**	**84.4**	-
Veggies	**98.8**	**99.1**	**88.7**	-	**98.3**	99.2	**86.2**	-	**99.4**	99.0	**90.4**	-
Carbs	**99.2**	**99.2**	**24.3**	-	99.2	99.3	26.3	-	99.3	99.1	23.5	-
Dairy	**68.5**	**71.0**	**46.7**	-	**63.8**	**68.0**	**41.5**	-	**73.4**	**74.0**	**50.6**	-

^a^Bold numbers indicate a significant difference in trends (*p* < 0.05). ^b^Bold numbers indicate a significant gender difference in sociodemographic factors within each wave (*p* < 0.05)

### Trends in disability

Table 2 presents both the prevalence of disability over time after age standardization, and the trend of disability in the entire sample, as well as the gender differences in the disability trend by year. It was found that the prevalence rate of ADL disabilities was 15.3–17.3%; the prevalence rate of IADL disabilities was 36.0–43.5%; the prevalence rate of mobility disabilities was 49.5–56.6%, and the prevalence rate of frailty was 27.6–35.1%. However, with the exception of IADL-related disabilities, where the trend could not be estimated, the other types of disability showed a downward trend (${\chi }_{ADL}^{2}= -1.2$, ${\chi }_{Mobility}^{2}= -5.4$, ${\chi }_{Frailty}^{2}= -6.2$), where only ADL did not reach statistical significance. In addition, the prevalence of disabilities among women was significantly higher than that among men over the observation period (ADL: 17.4–20.5% vs. 11.7–15.3%; IADL: 41.1–53.5% vs. 30.5–33.5%; mobility: 58.3–68.2% vs. 39.2–45.1%; frailty: 34.5–43.4% vs. 20.1–26.5%). However, in terms of the trends, mobility (${\chi }_{men}^{2}= -2.9$, ${\chi }_{women}^{2}= -5.6$) and frailty (${\chi }_{men}^{2}= -4.1$, ${\chi }_{women}^{2}= -5.0$) exhibited a significant downward trend in both men and women. This result was the same as that shown in Table 3 using the logistic regression analysis (mobility: AOR_men_ = 0.9 (95% CI = 0.8–1.0), AOR_women_ = 0.8 (95% CI = 0.8–0.9), frailty: AOR_men_ = 0.8 (95% CI = 0.8–0.9), AOR_women_ = 0.8 (95% CI = 0.8–0.9)), where women exhibited a significant downward trend in IADL (${\chi }_{IADL}^{2}= -6.8$). These results were the same as those shown in Table 3 (IADL: AOR_women_ = 0.8 (95% CI = 0.7–0.9)). The prevalence of each disability in both sexes did thus not change statistically significantly over the years.

**Table 2:** Standardized prevalence of disability in men and women, trend analysis 2001–2013

Disability	Total				Men^a^				Women^a^				χ^2^ for trend			Trend difference
	2001	2005	2009	2013	2001	2005	2009	2013	2001	2005	2009	2013	Total	Men	Women	Gender*Year
Limitations in ADL	17.3	15.4	17.5	15.3	**15.3**	**11.7**	**13.7**	**13.2**	**19.4**	**19.0**	**20.5**	**17.4**	-1.17	-0.78	-1.08	1.24
Limitations in IADL	-	43.5	-	36.0	-	**33.5**	-	**30.5**	-	**53.5**	-	**41.1**		-1.72	**-6.84**	
Limitations in Mobility	-	56.6	52.1	49.5	-	**45.1**	**39.2**	**39.7**	-	**68.2**	**61.9**	**58.3**	**-5.42**	**-2.87**	**-5.58**	0.07
Frailty	-	35.1	30.8	27.6	-	**26.5**	**21.7**	**20.1**	-	**43.4**	**37.9**	**34.5**	**-6.21**	**-4.05**	**-5.00**	0.15

“-”means there were different questions in this wave compared with other waves, so no analysis was conducted. ^a^Bold numbers mean there were significant gender differences in disability factors within each wave (*p* < 0.05)

**Table 3:** Adjusted Odds Ratios (AOR) and 95% Confidence Intervals (CI) from Binary Logistic Regression Analyses Predicting ADL, IADL, Mobility, and Frailty in Men and Women

Characteristic	Men (*N* = 2,038)								Women (*N* = 2,235)								
	Limitations in ADL		Limitations in IADL		Limitations in Mobility		Frailty		Limitations in ADL			Limitations in IADL		Limitations in Mobility		Frailty	
	AOR	95%CI	AOR	95%CI	AOR	95%CI	AOR	95%CI	AOR		95%CI	AOR	95%CI	AOR	95%CI	AOR	95%CI
Sociodemographic																	
Age group (years, ref = 65–69)																	
70–74	0.94	0.53–1.69	1.29	0.88–1.89	**1.46**	**1.12–1.92**	1.34	0.92–1.93	0.94		0.61–1.47	**1.61**	**1.20–2.15**	**1.42**	**1.13–1.78**	**1.40**	**1.06–1.86**
75–79	1.58	0.92–2.72	**1.98**	**1.36–2.87**	**2.31**	**1.75–3.05**	**1.94**	**1.34–2.81**	**2.20**		**1.45–3.34**	**2.71**	**1.98–3.71**	**2.72**	**2.07–3.57**	**2.08**	**1.53–2.81**
80–84	**1.97**	**1.10–3.54**	**2.62**	**1.71–4.00**	**3.40**	**2.44–4.76**	**2.14**	**1.40–3.26**	**3.37**		**2.07–5.47**	**3.75**	**2.51–5.60**	**4.21**	**2.84–6.23**	**2.69**	**1.84–3.95**
85 +	**2.47**	**1.19–5.10**	**5.08**	**2.89–8.90**	**6.51**	**3.95–10.72**	**2.63**	**1.51–4.60**	**4.58**		**2.51–8.35**	**7.80**	**4.48–13.56**	**5.31**	**2.96–9.53**	**3.76**	**2.22–6.38**
Living alone	0.69	0.36–1.31	**0.47**	**0.29–0.77**	1.02	0.71–1.47	1.14	0.72–1.79	0.74		0.46–1.19	**0.51**	**0.35–0.75**	1.07	0.79–1.47	0.92	0.65–1.28
Married/Partnered	**0.63**	**0.40–0.98**	0.75	0.54–1.04	0.85	0.65–1.12	0.85	0.60–1.19	0.92		0.67–1.28	0.79	0.62–1.01	0.91	0.74–1.12	0.89	0.70–1.13
Level of education (ref = High)																	
Low	0.91	0.50–1.67	**1.98**	**1.25–3.14**	1.31	0.94–1.82	1.24	0.80–1.92	1.66		0.47–5.90	**2.86**	**1.19–6.90**	1.28	0.72–2.29	1.89	0.82–4.37
Medium	0.68	0.30–1.55	1.24	0.68–2.25	0.77	0.50–1.21	0.85	0.47–1.53	0.91		0.20–4.22	1.33	0.46–3.82	0.92	0.45–1.91	1.52	0.57–4.07
Health condition																	
Metabolic disease	**1.62**	**1.08–2.43**	**1.79**	**1.35–2.37**	**1.52**	**1.23–1.88**	**1.62**	**1.23–2.14**	**1.47**	**1.05–2.05**		**1.33**	**1.04–1.69**	**1.45**	**1.19–1.78**	**1.55**	**1.22–1.97**
Heart disease	1.18	0.74–1.89	1.29	0.92–1.80	**1.38**	**1.05–1.80**	**1.57**	**1.13–2.18**	0.83	0.58–1.20		1.19	0.90–1.58	**1.74**	**1.33–2.27**	**1.92**	**1.47–2.51**
Lung disease	**1.82**	**1.11–2.98**	**1.96**	**1.34–2.85**	**1.96**	**1.41–2.74**	**1.50**	**1.01–2.22**	**2.13**	**1.31–3.45**		1.53	0.99–2.34	**1.64**	**1.07–2.50**	**1.55**	**1.01–2.39**
Cataracts	0.94	0.62–1.41	0.94	0.71–1.26	1.21	0.97–1.50	1.00	0.75–1.34	1.33	0.99–1.79		1.18	0.94–1.49	**1.40**	**1.15–1.70**	**1.27**	**1.02–1.59**
Hearing impairment	0.91	0.32–2.60	**2.40**	**1.19–4.82**	1.23	0.65–2.34	1.31	0.62–2.75	2.49	0.96–6.46		2.49	0.97–6.41	1.16	0.44–3.06	1.15	0.43–3.07
Cognitive impairment	**3.64**	**2.38–5.57**	**3.12**	**2.21–4.38**	**1.95**	**1.45–2.63**	**1.96**	**1.38–2.78**	1.29	0.94–1.78		**1.93**	**1.49–2.49**	1.08	0.84–1.37	1.19	0.92–1.53
Behavioral factors																	
BMI (ref = Normal)																	
Underweight	1.24	0.62–2.51	1.22	0.71–2.10	1.04	0.65–1.67	1.65	0.96–2.81	1.57	0.87–2.81		1.26	0.75–2.11	0.82	0.52–1.31	0.85	0.51–1.41
Overweight	0.98	0.62–1.55	0.96	0.70–1.32	**1.27**	**1.00–1.62**	0.77	0.56–1.06	0.99	0.68–1.44		1.21	0.92–1.59	**1.32**	**1.05–1.66**	1.00	0.77–1.31
Obese	1.08	0.63–1.86	1.01	0.70–1.47	**1.92**	**1.45–2.54**	1.13	0.79–1.62	**1.58**	**1.09–2.28**		**1.63**	**1.22–2.17**	**2.00**	**1.55–2.58**	1.20	0.91–1.59
Exercise	**0.34**	**0.23–0.51**	**0.70**	**0.53–0.91**	**0.70**	**0.57–0.86**	**0.39**	**0.30–0.50**	**0.51**	**0.37–0.70**		0.8	0.63–1.01	**0.77**	**0.63–0.94**	**0.42**	**0.33–0.52**
Falls	**2.84**	**1.88–4.29**	**2.68**	**1.96–3.68**	**2.12**	**1.61–2.80**	**2.15**	**1.56–2.96**	**2.10**	**1.55–2.85**		**1.46**	**1.13–1.88**	**1.77**	**1.40–2.25**	**1.68**	**1.31–2.15**
Social engagement	**0.42**	**0.26–0.66**	**0.47**	**0.35–0.63**	0.82	0.66–1.01	**0.28**	**0.21–0.38**	**0.39**	**0.27–0.55**		**0.49**	**0.39–0.62**	**0.64**	**0.53–0.78**	**0.32**	**0.26–0.41**
Depressive symptoms	**2.21**	**1.36–3.60**	**2.43**	**1.68–3.50**	**2.82**	**1.99–4.00**	**8.50**	**5.82–12.42**	**2.72**	**1.89–3.92**		**3.61**	**2.67–4.90**	**3.37**	**2.37–4.78**	**10.89**	**7.65–15.50**
Diet																	
Tea or coffee	**0.58**	**0.40–0.86**	**0.72**	**0.55–0.95**	**0.73**	**0.59–0.91**	**0.57**	**0.43–0.74**	**0.70**	**0.51–0.96**		0.89	0.70–1.12	**0.68**	**0.56–0.83**	**0.66**	**0.53–0.83**
Protein	**0.35**	**0.16–0.80**	0.87	0.40–1.90	1.31	0.71–2.41	1.02	0.49–2.15	0.68	0.41–1.13		1.12	0.70–1.81	0.88	0.61–1.28	0.73	0.49–1.09
Veggies	0.99	0.51–1.95	1.19	0.65–2.17	0.96	0.63–1.46	0.86	0.52–1.42	0.83	0.43–1.60		0.89	0.48–1.64	0.96	0.59–1.58	0.72	0.43–1.21
Carbs	1.17	0.75–1.82	**3.72**	**2.63–5.26**	**1.42**	**1.12–1.78**	**1.47**	**1.08–1.99**	1.12	0.80–1.58		**6.11**	**4.60–8.12**	**1.42**	**1.15–1.75**	**1.41**	**1.11–1.80**
Dairy	1.36	0.92–2.00	**1.36**	**1.03–1.78**	1.02	0.83–1.26	1.01	0.77–1.33	**1.49**	**1.09–2.04**		1.12	0.89–1.43	1.16	0.95–1.41	**1.34**	**1.06–1.68**

Bold numbers indicate a significant difference (*p* < 0.05)

### Disability-related factors

Table 3 shows the results of the logistic regression assessing the gender-related risks and protective factors for each of the disabilities related to ADL, IADL, mobility, and frailty. In terms of ADL disability, cognitive impairment was found to be the most relevant risk factor for ADL disabilities in older men. Cognitive impairment had a 3.6-fold correlation with ADL disabilities in men (AOR_cognitive impairment_ = 3.6, 95% CI = 2.4–5.6). In contrast, the most relevant factor contributing to ADL disabilities is women was found to be age. ADL disabilities in women aged 75–59, 80–84, and over 85 were 2.2 times (95% CI = 1.5–3.3), 3.4 (95% CI = 2.1–5.5), and 4.6 ( 95% CI = 2.5–8.4) that of women aged 65–69, respectively. Among the risk factors related to healthy behavior, both falls and depressive symptomatology were significant factors related to ADL disabilities in both men and women. In men and women, falling was, respectively 2.8 (95% CI = 1.9–4.3) and 2.1 (95% CI = 1.6–2.9) times higher in those with ADL disabilities. In both men and women, depressive symptomatology was 2.2 times (95% CI = 1.4–3.6) and 2.7 (95% CI = 1.9–3.9) more likely to be associated with ADL disabilities. Obesity (BMI ≧ 27) was more related to ADL disabilities in women (AOR_obesity_ = 1.6, 95% CI = 1.1–2.3), than in men (AOR_obesity_ = 1.1, 95% CI = 0.6–1.9). In addition, in terms of disease factors, except for the fact that cognitive impairment could significantly predict disabilities in men, the common disease risk factors affecting ADL disabilities in both men and women included lung disease and metabolic diseases. Lung disease was 1.8 times (95% CI = 1.1–3.0) and 2.1 (95% CI = 1.3–3.5) more likely to be associated with ADL disabilities in both men and women, respectively, while metabolic disease was 1.6 times (95% CI = 1.1–2.4) and 1.5 (95% CI = 1.1–2.1) more likely to be associated with ADL disabilities in both men and women, respectively. In terms of protective factors, exercise, social engagement, and tea or coffee intake habit were found to be significant predictors in the data spanning 13 years covering a total of 10,896 representative subjects across Taiwan who were found to be less likely to report ADL disabilities. Although these protective factors were controlled, sociodemographic and disease variables still existed. Among them, the independent protective factors related to ADL disabilities among women included the following: social engagement (AOR_Social Engagement_ = 0.4, 95% CI = 0.3–0.6), exercise (AOR_Exercise_ = 0.5, 95% CI = 0.4–0.7), and tea or coffee intake habit (AOR_Tea or coffee_ = 0.7, 95% CI = 0.5–1.0), while the independent protective factors related to ADL disabilities among men were as follows: exercise (AOR_Exercise_ = 0.3, 95% CI = 0.2–0.5), eggs/beans/fish/meat consumption (AOR_Eggs/Beans/Fish/Meat_ = 0.4, 95% CI = 0.2–0.8), social engagement (AOR_Social Engagement_ = 0.4, 95% CI = 0.3–0.7), tea or coffee intake habit (AOR_Tea or Coffee_ = 0.6, 95% CI = 0.4–0.9), and being married (AOR_Married_ = 0.6, 95% CI = 0.4–1.0).

In terms of independent protective and risk factors related to IADL disabilities, we found that age, especially in the case of those aged 85 or above, is the most important risk factor related to IADL disabilities in both men and women, as it increases the prevalence of disabilities in men and women by 5.1 (95% CI = 2.9–8.9) and 7.8 (95% CI = 4.5–13.6) times, respectively. In addition, time differences were also found in the effects of age on men and women, where men at age 75 or above (AOR_75-79 years old_ = 2.0, 95% CI = 1.4–2.9) and women at age 70 or above (AOR_70-75 years old_ = 2.7, 95% CI = 1.9–3.7) exhibited significant independent correlations with IADL disabilities. Among the risk factors related to healthy behavior, the intake of carbohydrates was second only to the age factor, and it was the most relevant factor that predicted IADL disabilities independently in men and women. The intake of carbohydrates was 6.1 times more likely to lead to IADL disabilities in women (95% CI = 4.6–8.1) and 3.7 times more likely to be correlated with IADL disabilities in men (95% CI = 2.6–5.3). In addition, factors significantly related to IADL disabilities also included depressive symptoms and obesity in women, where depressive symptomatology was 2.4 (95% CI = 1.7–3.5) and 3.6 (95% CI = 2.7–4.9) times more likely to be significantly correlated with IADL disabilities in men and women, respectively, while obesity was a specific factor related to IADL disabilities in elderly women. The prevalence of IADL disabilities among the obese elderly women was 1.6 times higher than that for women of normal weight (95% CI = 1.2–2.2). Among other diseases and behavioral factors, cognitive impairment, falls, and metabolic diseases were significantly associated with IADL disabilities in all genders. Metabolic disease (based on the respondent having been confirmed by a doctor to have hypertension, diabetes, or hyperlipidemia) led to a similar degree of IADL disabilities in both men and women, where metabolic diseases were 1.8 times (95% CI = 1.4–2.4) and 1.3 (95% CI = 1.0–1.7) more likely to be correlated with IADL disabilities in men and women, respectively. Meanwhile, cognitive impairment and falling were far more important for men than women, and cognitive impairment was 3.1% (95% CI = 2.2–4.4) and 1.9 (95%CI = 1.5–2.5) times more likely to be correlated with IADL disabilities in men and women, respectively, while falling was 2.7 times (95% CI = 2.0–3.7) and 1.5 (95% CI = 1.1–1.9) more likely to be correlated with IADL disabilities in both men and women, respectively. In addition, hearing problems and lung disease were unique independent factors correlated with IADL disabilities only in men, where hearing problems were 2.4 times more likely to be related to IADL disabilities in men (95% CI = 1.2–4.8), and lung disease was 2.0 times more likely to be related to IADL disabilities in men (95% CI = 1.3–2.9). The correlations with hearing problems and lung disease with IADL disabilities in women was not significant, which may have been due to the definition of hearing problems in this study. Finally, the common protective factors for men and women against IADL disability included living alone and social engagement. Living alone significantly protected men and women by about 53% (AOR_Living Alone_ = 0.5, 95% CI = 0.3–0.8) and 49% (AOR_Living Alone_ = 0.5, 95% CI = 0.4–0.8) against IADL disabilities, respectively. Social engagement significantly protected men and women by about 53% (AOR_Living Alone_ = 0.5, 95% CI = 0.3–0.6) and 51% (AOR_Living Alone_ = 0.5, 95% CI = 0.4–0.6) against IADL disabilities, respectively. In addition, the protective factors against IADL disabilities in men also included exercise (AOR_Exercise_ = 0.7, 95% CI = 0.5–0.9) and tea or coffee intake (AOR_Tea or Coffee_ = 0.7, 95% CI = 0.6–1.0).

In the regression analysis of the independent protective and risk factors related to upper and lower limb disabilities, it was found that the independent predictor most relevant to the level of upper and lower limbs disabilities in both men and women was age. In men and women, the risks of upper and lower limb disabilities in those aged 85 or more, 80–84, 75–79, and 70–74 were 5.3–6.5 times greater (Men: AOR 85 or older = 6.5, 95% CI = 4.0–10.7; women: AOR 85 or older = 5.3, 95% CI = 3.0–9.5), 3.4–4.2 times greater (men: AOR80-84 = 3.4, 95% CI = 2.4–4.8; women: AOR80-84 = 4.2, 95% CI = 2.8–6.2), 2.3–2.7 times greater (men: AOR75-79 = 2.3, 95% CI = 1.8–3.1; women: AOR75-79 = 2.7, 95% CI = 2.1–3.6), and 1.4–1.5 times greater (men: AOR70-74 = 1.5, 95% CI = 1.1–1.9; women: AOR70-74 = 1.4, 95% CI = 1.1–1.8) than those in individuals ranging in age from 65–69. In addition to age, other sociodemographic factors, including living alone, marriage, and education level, were not found to be significantly related to upper and lower limb disabilities in either men or women. Among the risk factors associated with healthy behavior, depressive symptoms (Men: AOR_Depressive Symptomatology_ = 2.8, Women: AOR_Depressive Symptomatology_ = 3.4), being overweight (Men: AOR_Overweight_ = 1.3, Women: AOR_Overweight_ = 1.3), being obese (Men: AOR_obesity_ = 1.9, Women: AOR_obesity_ = 2.0), falling (Men: AOR_Fall_ = 2.1, Women: AOR_Fall_ = 1.8), and a high carbohydrate diet (Men: AOR_Carbohydrate_ = 1.4, Women: AOR_Carbohydrate_ = 1.4) were found to be significantly related to upper and lower limb disabilities. In terms of disease factors, risk factors for upper and lower limb disabilities included lung disease (Men: AOR_Lung Disease_ = 2.0, Women: AOR_Lung Disease_ = 1.6), metabolic diseases (Men: AOR _Metabolic disease_ = 1.5, Women: AOR _Metabolic disease_ = 1.5), and heart disease (Men: AOR_Heart disease_ = 1.4, Women: AOR_Heart disease_ = 1.7). Meanwhile, cognitive impairment in men (AOR_Cognitive Impairment_ = 2.0, 95% CI = 1.4–2.6) and cataracts in women (AOR_Cataract_ = 1.4, 95% CI = 1.2–1.7) were also related to upper and lower limb disabilities. However, hearing problems were not found to be significantly related to upper and lower limb disabilities in either gender. Finally, the common protective factors against upper and lower limb disabilities in men and women included exercise and tea or coffee intake. Exercise significantly protects men and women, respectively, by approximately 30% (AOR_Exercise_ = 0.7, 95% CI = 0.6–0.9) and 23% (AOR_Exercise_ = 0.8, 95% CI = 0.6–0.9) against upper and lower limb disabilities. Tea or coffee intake was found to significantly protect men and women, respectively, by about 27% (AOR_Tea or Coffee_ = 0.7, 95% CI = 0.6–0.9) and 32% (AOR_Tea or Coffee_ = 0.7, 95% CI = 0.6–0.8) against upper and lower limb disabilities. In addition, another factor protecting women against upper and lower limb disabilities included social engagement (AOR_Social Engagement_ = 0.6, 95% CI = 0.5–0.8).

**Fig. 1:**
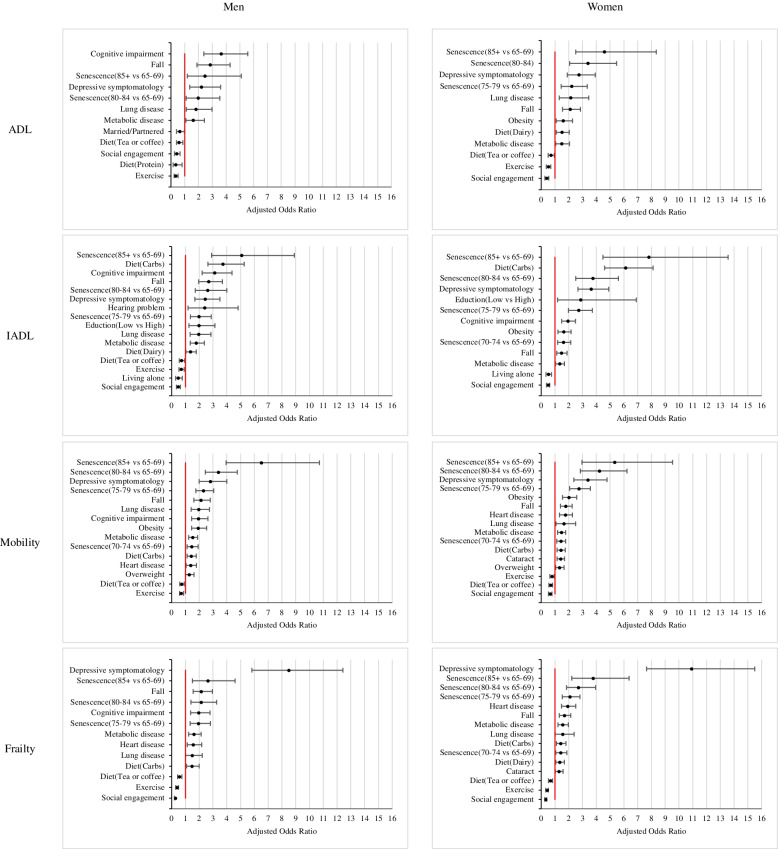
The adjusted odds ratio of risk of disability and protective factors for men and women

Among the predictive factors for frailty, depressive symptomatology was the most relevant risk factor for both men and women. Depression was 8.5 times (95% CI = 5.8–12.4) and 10.9 (95% CI = 7.7–15.5) more likely to be related to frailty, respectively, for each gender. Age, especially the age of 85 and above, was second only to depressive symptomatology, which is also a factor that affected frailty levels in both men and women. In addition, there was also a time difference in terms of the influence of age on frailty in men and women. The age of 75 or above in men (AOR_75-79_ = 1.9, 95%CI = 1.3–2.8) and the age of 70 or above in women (AOR_70-75_ = 1.4, 95%CI = 1.1–1.9) were significantly associated with frailty. In addition to age, other sociodemographic factors, including living alone, marriage, and education level, were not found to be significantly related to frailty in either gender. Among the risk factors related to healthy behavior, in addition to depressive symptomatology, falling (Men: AOR_Fall_ = 2.2, Women: AOR_Fall_ = 1.7) and a diet based on carbohydrates (Men: AOR_Carbohydrate_ = 1.5, Women: AOR_Carbohydrate_ = 1.3) were also significantly related to frailty, and a diet based on dairy products (AOR_Milk_ = 1.3) was significantly correlated with frailty in women. Among disease factors, metabolic disease Men: AOR_Three-Highs_ = 1.6, Women: AOR_Three-Highs_ = 1.6), heart disease (Men: AOR_Heart disease_ = 1.6, Women: AOR_Heart disease_ = 1.9), and lung disease (Men: AOR_Lung Disease_ = 1.5, Women: AOR_Lung Disease_ = 1.6) were related to frailty in all genders. Meanwhile, cognitive impairment in men (AOR_Cognitive Impairment_ = 2.0, 95% CI = 1.4–2.8) and cataracts in women (AOR_Cataract_ = 1.3, 95% CI = 1.0–1.6) were also related to frailty, but hearing problems were not found to be significantly related to frailty in either gender. Finally, the common protective factors for men and women against frailty included social engagement, exercise, and tea or coffee intake, in that order. Social engagement significantly protected men and women by about 72% (AOR_Social Engagement_ = 0.3, 95% CI = 0.2–0.4) and 68% (AOR_Social Engagement_ = 0.3, 95% CI = 0.3–0.4) against frailty. Exercise significantly protected men and women by approximately 61% (AOR_Exercise_ = 0.4, 95% CI = 0.3–0.5) and 58% (AOR_Exercise_ = 0.4, 95% CI = 0.3–0.5) against frailty, respectively. Tea or coffee intake habit significantly protected men and women by about 43% (AOR_Tea or Coffee_ = 0.6, 95% CI = 0.4–0.7) and 33% (AOR_Tea or Coffee_ = 0.7, 95% CI = 0.5–0.8) against frailty, respectively. Figure 1 illustrate the independent impacts of distinct biopsychosocial correlates for each disability outcome.

## Discussion

By analyzing nation-wide data on disability in older adults in Taiwan, this study reveals distinct risk and protective factors associated with frailty, mobility, IADL, and ADL in older men and women in Taiwan.

The common risk factors for both men and women in ADL disability included higher age (men over 80 years old, women over 75 years old), depressive symptomatology, and falls. For men, cognitive impairment was also found to be a risk factor, and for women, lung disease was also a risk factor. Common risk factors for men and women in IADL included higher age (men over 80 years old, women over 75 years old), depressive symptomatology, and consumption of carbohydrates. For men, cognitive and hearing impairments were also risk factors, and for women, low education level was also one of the risk factors. The common risk factors for men and women in terms of mobility included higher age (men and women over 70 years old) and depressive symptomatology. For men, lung disease was also one of the risk factors related to mobility, and for women, obesity and falling were also among the risk factors. Common risk factors for men and women in terms of frailty included higher age (men over 75 years old, women over 70 years old), depressive symptomatology, and falls.

Protective factors associated with disability in all genders were also found. In terms of protective factors for ADL, the common factors included exercise, social engagement, and eating habits. It was found that eating eggs and fish protects men, while drinking tea and coffee protects women. Regarding the protective factors against IADL disabilities, the common factors were social engagement and living alone in both men and women. In terms of protective factors related to mobility, the common factors for men and women included social engagement, exercise, and diet (especially tea and coffee). In terms of the protective factors against frailty, the common factors for men and women included social engagement, exercise, and diet (tea and coffee consumption).

The results indicating that the prevalence of disabilities in women is greater than that in men were consistent with the findings of studies in other countries. An analyzed national health survey data in Spain from 2001 to 2007 and found that the prevalence of disabilities in ADL, IADL, and mobility in women was higher than that in men [39], which was the same as the findings in Taiwan. The risk factors for ADL and IADL disabilities were found to be multiple chronic diseases, advanced age (over the age of 84), lack of exercise, low education level, and obesity (women). The risk factors for disabilities in mobility included higher age, lack of exercise, and two or more chronic diseases, which was similar to the situation in Taiwan. The difference is that the prevalence of disability in Spain had a significant upward trend, compared with a significant decrease in Taiwan [39]. Similar results have also been observed in studies in the United States. Women live longer than men. If the average life expectancy is divided into active average life expectancy and disabled life expectancy, it was found that the active average life expectancy and disabled life expectancy are both longer in women than in men. This infers that disability is associated with higher rates of chronic disease, lower muscle strength and bone density, obesity, and sedentary habits in women [40, 41].

Depression was also found to be a common risk factor for the four disability items examined in the present study. Disability and depression are interrelated [42]. Depression can be used to predict the likelihood of disabilities in healthy older adults. According to an analysis conducted by Penninx et al. comparing people with and without depressive symptoms, people with depression experienced a significant decline in physiological functioning over four years of tracking [43]. Similar findings have also been found in other related studies. A systematic review indicated that there is a reciprocal causation between depression and frailty syndrome and they share common pathophysiological mechanisms, including local inflammation and hormone changes related to the hypothalamic–pituitary–adrenal (HPA) axis [44]. On the other hand, some studies have found that disabilities can increase the likelihood of depression, especially when a person is bedridden for more than two weeks, and can significantly and strongly predict the occurrence of depression [45]. Other studies have also inferred these connections. It is not that there is a direct causal relationship between depression and disability, but that there is a third interference factor (confounding factor). The most common interfering factor is a physical disorder. Since disease can cause depression and disability at the same time, it is indirectly related to depression and disability [42].

Excessive BMI (obesity) is also one of the factors related to disability. In the elderly, sarcopenic obesity is increasing. Sarcopenic obesity represents a decrease in the proportion of muscle and an increase in body fat. This condition has more adverse effects than simple obesity or muscle loss, and also increases the risk of three types of disability: ADL, IADL, and frailty [46]. However, some studies have pointed out that a BMI indicating that an individual is overweight has a protective effect on disability as compared to normal or obese groups [47]. However, according to the overall body of literature on this topic, the ratio of muscle mass can predict disability more directly than the BMI, where a lower muscle mass is an indicator of poorer mobility performance and a greater chance of disability. From this study, maintaining muscle mass through exercise is also one of the protective factors that will prevent disability.

Another finding of this study is the effect of dietary habits on disability. The analytical results showed that eating carbohydrates is a common risk factor for IADL disabilities in both men and women, while drinking tea and coffee is a protective factor. The most common carbohydrate in Taiwan is rice. In both South Korea and East Asia, two different dietary patterns have been analyzed: a traditional dietary pattern and a modified traditional dietary pattern. The biggest difference between the two is the rice intake. The traditional dietary pattern is mainly based on rice, while the modified dietary pattern is mainly based on a lower proportion of rice and a higher proportion of vegetables, fruits, and dairy products. It was found that the adjusted diet had a high correlation with a decline in ADL in men and the decline in both ADL and IADL in women [48]. Another Japanese study pointed out that eating rice increases the risk of Japanese women developing type 2 diabetes [49]. Adding pinto beans, kidney beans, and black beans to rice can reduce the glycemic index more effectively than simply eating rice, and it is beneficial to controlling type 2 diabetes [50]. Diabetes can cause chronic inflammation in the body, decrease muscle mass, and even lead to other chronic diseases such as chronic kidney disease and cerebrovascular disease. All these increase the risk of disability [51]. Tomata et al. found that drinking green tea can help reduce the risk of disability. Polyphenols in green tea can improve muscle strength, improve common sarcopenia in the elderly, and prevent diseases related to disability (such as stroke and dementia). At the same time, green tea also plays an important role in Japanese society [52]. The findings of the present study also showed that social participation is a protective factor against disabilities. The common ingredient in tea and coffee is caffeine. Caffeine reduces the damage to the brain caused by Aβ plaque, a harmful substance in the brain. Aβ plaque is also the most important pathological feature of Alzheimer’s disease. In animal experiments, caffeine has been shown to promote improved memory and reverse the pathophysiology of Alzheimer’s disease. Drinking caffeine-containing substances can thus help maintain cognitive function [53].

The limitations of this study include the fact that the sample lacks an institutionalized population. Generally, elderly people living in institutions have poor physical and cognitive functions. The absence of samples residing in institutions may have led to underestimation of the disability ratio. In addition, it was not comprehensive enough to measure frailty simply using a self-report questionnaire, especially for measurements of weakness, which would be more ideally assessed based on a handgrip strength measurement. In addition, due to being limited to the use of secondary data, the assessment of food intake was classified by frequency alone. In our study, it was not possible to distinguish between people who eat a lot at once from those who eat small amounts frequently. Thus, researchers are encouraged to further explore details of food intake in this line of research. In addition, the risk and protective correlates were measured along with the disability data; thus, a causal relationship could not be confirmed from the findings of the present study.

Despite the above limitations, by using national level data from Taiwan, trends in risk and protective factors of disability in older adults during the past decade were revealed. The results not only provide empirical evidence that exercise, social engagement, and diet are important protective factors against ADL disabilities, mobility disabilities, and frailty in both men and women, it extends the current understanding that cognitive impairment and lung disease are important correlates for ADL disabilities in men, and depressive symptomatology is a common and high risk factor for higher IADL disabilities, mobility disabilities, and frailty in both men and women. In addition, a high carbohydrate diet was associated with higher levels of IADL disabilities in both men and women. We believe the findings from this study provide insights that may help guard against functional decline in older adults. Specifically, it supports the importance of the beneficial roles of exercise and social engagement in guarding against disability. In addition, a healthy diet, including lower carbohydrates, higher protein, and tea consumption, was shown to guard against disability in older adults in the present study. Attention should be also paid to cognitive maintenance and prevention of depressive symptomatology to help prevent disabilities in the older population.

## Data Availability

The data that support this study are available from the Health Data Science Center, but restrictions apply to the availability of these data, which were used under license for the current study, and so are not publicly available. Data are however available from the authors upon reasonable request and with permission of the Health Data Science Center.

## Abbreviations

ADL: Activities of Daily Living
IADL: Instrumental Activities of Daily Living
NHIS: National Health Interview Survey
PPS: Probability Proportional to Size
MMSE: Mini-mental State Examination
MCI: Mild cognitive impairment
CES-D: Center for Epidemiologic Studies Depression Scale
BMI: Body mass index
